# A Linguistic Analysis of Health Literacy Demands of Chronic Kidney Disease Patient Education Materials

**DOI:** 10.3928/24748307-20171227-01

**Published:** 2018-01-23

**Authors:** Suzanne Morony, Angela C. Webster, Rachelle Buchbinder, Suzanne Kirkendall, Kirsten J. McCaffery, Rosemary Clerehan

## Abstract

**Background:**

Instruments to assess the quality and comprehensibility of printed patient education materials may lack proper consideration of how readers derive meaning from text. The Evaluative Linguistic Framework (ELF) considers how factors that influence readers' expectations about health care texts also affect their ability to understand them. The ELF has demonstrated value in improving the quality of patient materials about medication, consent, and self-reported questionnaires, but has not yet been used to evaluate a corpus of patient education materials about chronic disease self-management.

**Objective:**

This study sought to apply the ELF to examine specific elements of printed self-management patient education materials for chronic kidney disease (CKD) not captured by other tools.

**Methods:**

From a previously published systematic review, we identified 14 patient education materials (eight self-management, six diet and nutrition) for people with CKD. We used the ELF to identify the different ways the text could be structured, its intended purpose, the relationship established between reader and writer, presence of signposting, its complexity and technicality of language, and factual content.

**Key Results:**

Our analysis identified nine possible structural units, of which “introducing the problem” and “instructing the reader to self-manage” were common to all materials. However, there was no consistency or common sequence to these units of text. The intended readership and aims of the author(s) were not always clear; many materials made assumptions about what the reader knew, the language was often complex and dense, and the meta-discourse was sometimes distracting.

**Conclusions:**

Our analysis suggests CKD document developers can benefit from a theoretically grounded linguistic tool that focuses on the intended audience and their specific needs. The ELF identified structural units of text, aligned with rhetorical elements that can be uniformly applied for developing self-management education materials for CKD, and provided checks for language complexity. Further work can determine its usefulness for other (e.g., electronic) formats and other chronic diseases. **[*HLRP: Health Literacy Research and Practice*. 2018;2(1):e1–e14.]**

**Plain Language Summary:**

Helping patients make meaning from information about their condition is a key goal of health care organizations. We analyzed chronic kidney disease patient education materials on self-management using the Evaluative Linguistic Framework. The purpose and intended audience were frequently unclear. We identified nine structural units of text that may assist information providers to plan and structure content.

Printed patient education materials offer clinicians a way to present salient information that can be reviewed by patients in their own time, but their effectiveness depends on how appropriate they are for the patient group. People with chronic kidney disease (CKD), a complex and progressive condition influenced by lifestyle factors (Lopez-Vargas, Tong, Howell, & Craig, 2016), have repeatedly requested quality information on self-management (Tong et al., 2008). Limited health literacy is common among people with CKD (Fraser et al., 2013; Taylor et al., 2017), and is associated with a higher disease burden, higher health care costs, and poorer health outcomes (Berkman, Sheridan, Donahue, Halpern, & Crotty, 2011). In a recent systematic review of English-language CKD patient information materials, most of what was analyzed (*n* = 80) had a reading level supposedly too high for the average patient (Morony, Flynn, McCaffery, Jansen, & Webster, 2015). Examination of a subset of 26 lifestyle-specific materials using the Suitability Assessment of Materials (SAM) (Doak, Doak, & Root, 1996) and Patient Education Materials Assessment Tool for Printable Materials (PEMAT-P) (Shoemaker, Wolf, & Brach, 2013) revealed that use of visual aids was generally poor and the required actions to take were often unclear (Morony, McCaffery, Kirkendall, Jansen, & Webster, 2017). Creating suitable patient education materials for particular populations is an ongoing challenge for health care practitioners and organizations.

Although tools for developing (e.g., CDC Clear Communication Index [Centers for Disease Control and Prevention, 2013]), and evaluating (e.g., SAM [Doak et al., 1996], PEMAT [Shoemaker et al., 2013]) patient materials consider the “average” or “low-literacy” user and can guide improvements to presentation of important information, for materials to be most effective they must be appropriate for “this” user. An approach that seeks explicitly to assess both “comprehensibility” and “usability” with respect to a specific audience's needs is the Evaluative Linguistic Framework (ELF), which is grounded in linguistic theory and concerned with how readers make meaning from text (Clerehan, Buchbinder, & Moodie, 2005). The ELF has demonstrated value for improving the quality and comprehensibility of medical information (Clerehan & Buchbinder, 2006; Hirsh, Clerehan, Staples, Osborne, & Buchbinder, 2009) and consent forms (Sand, Eik-Nes, & Loge, 2012), and evaluating the comprehensibility and cross-cultural suitability of self-report questionnaires (Clerehan, Guillemin, Epstein, & Buchbinder, 2016; Petkovic et al., 2015). It has not previously been used to investigate a corpus of chronic disease self-management education materials.

The ELF draws on the Systemic Functional Linguistics theory to assess patient information materials within the dual contexts of culture (involving social practices, knowledge, and values that dictate the type [or “genre”] of the text) and situation (what is being talked about, who is involved, and the channel of communication) (Halliday & Matthiessen, 2014). The interaction between text and context is where readers construct meaning (Clerehan et al., 2005). The extent to which a text is appropriate for the context of culture (what a reader would expect from a given type of text) and the context of situation (the subject matter, the acknowledged or unacknowledged identities of author and reader, and the way the text is conveyed, such as book chapter or brochure) affects its comprehensibility and usefulness for readers. The ELF was developed to evaluate health care texts and is comprised of 9 items and 22 assessment probes. These consider the generic structure of the document, its stated purpose, the relationship established between writer and reader, the use of signposting, the technicality of language, the density of “content” words in the text, and factual content.

Knowing who the user is and how they make meaning from text is important in CKD, because as the condition progresses, self-management activities change so information needs change with them. Furthermore, lifestyle is directly influenced by culture, including cultural norms about food and exercise. Self-management in CKD is complex and requires management of blood pressure, weight, cholesterol, physical activity, diet, fluids, and medications, as well as navigation of health care systems (Devraj & Gordon, 2009). We limit our study to two related topics: (1) self-management and (2) diet and nutrition. These topics were selected because they are under a patient's control and can significantly influence quality of life, and because materials within these two fields should cover similar subtopics (nutrition is an element of self-management). Self-management is important in many chronic conditions, so some findings may generalize.

This study aims to apply a functional linguistic approach to the examination of CKD patient education materials targeting lifestyle self-management. It builds on previous work by considering how specific features of the texts can assist readers to make meaning, thus demonstrating the unique contribution of the ELF to the analysis (and by extension development) of health care discourse.

## Methods

From our previous analyses of CKD patient education materials (Morony et al., 2015; Morony et al., 2017), we identified 8 publications that focused on self-management and 15 that focused on diet and nutrition. To ensure materials were comparable for the present study, we included dietary materials only if they covered a range of dietary factors relevant to CKD patients. Texts focusing on only one nutrient or mineral (e.g., protein, phosphate) were excluded (*n* = 6), as was general advice about how to read food labels (*n* = 1). Dining out guides (*n* = 2), which aimed to support patients to make food choices and did not educate about nutrient needs and restrictions (this knowledge was assumed), were similarly excluded. Our sample included eight self-management texts and six diet and nutrition texts. Two assessors (S.M. and S.K.) examined the CKD texts using the ELF assessment probes (**Table 1**), with contributions from all authors including the original developers of the ELF (R.C. and R.B.).

The ELF framework involves consideration of the following nine items. **Table 1** provides a description of each linguistic item and its assessment probes.

### Generic Structure

Different types of texts (within the same field or topic area) typically have a “generic structure” that makes sense for a particular audience. For example, an empirical scientific article typically has five key structural units or “moves” (i.e., abstract, introduction, method, results, discussion) of text that occur in a particular order and can vary somewhat according to discipline and journal conventions. It is a different genre from a recipe, although both have a “method” section. Readers usually will have expectations of which “moves” should be included, and what their sequence should be (Clerehan et al., 2005).

We examined the moves of the two sets of CKD patient education materials (self-management and diet) in separate analyses, and considered how frequently individual moves were present and if the order changed.

### Rhetorical Elements

The rhetorical function of a move is the purpose of the text in relation to the reader (e.g., to define, inform, instruct). If the function of the move is to instruct then it should be expressed as a clear command (e.g., “do not smoke”), whereas text given to inform should be conveyed as statements. We examined whether the rhetorical function of the text was consistent with the move.

### Writer-Reader Relationship

The identity of the author and reader, their relative status, and the implied relationship (the tenor) is indicated by forms of address. The audience of CKD patients using the materials may include those newly diagnosed, those who have been living with CKD for some time, and family members of people with CKD who may take responsibility for food shopping or preparation. Language may range from the collaborative and personal (e.g., “we will help you”) to the impersonal and directive (e.g., “patients should”). Patients have stated that tone (encouraging, reassuring) is important to them (Hirsh et al., 2009). We investigated whether the identity and expertise of the author, and the needs of the intended reader (such as their particular stage of CKD), were clear.

### Metadiscourse

Metadiscourse is text about the text. It does not add content but can indicate the purpose of the text and how readers should move around the text. When metadiscourse is absent, readers may not recognize the point of the material, whether it is aimed at them, and what they should do with it. We identified whether there was a clear description of the purpose of the material and also whether disclaimers (indications of what patients should not do with the materials) were present.

### Signposting

Headings act as signposts to assist readers in navigating the text by providing structure and foreshadowing what text will follow. Headings can be unhelpful if they are vague, overinclusive, or contain technical language. We looked for examples of helpful and unhelpful headings.

### Technicality of Vocabulary

The choice of vocabulary reflects the writer's expectations about the reader's level of understanding. We examined materials for choice of vocabulary, particularly the use of uncommon words or medical terms that were not explained.

### Lexical Density

The density of text indicates how closely packed the information is. It is typically calculated by dividing the number of content words by the number of clauses (Clerehan, et al., 2005). Lexical density can also be understood as the proportion of content-carrying words (nouns, verbs, adverbs, and adjectives) in a text or sentence (Eggins, 2004), expressed as a number out of 10. The average lexical density for spoken English is between 1.5 and 2, due to a high proportion of function words (i.e., grammatical words such as “and,” “the,” and “be”), compared with 3 to 6 for most written English. A lexical density of below 5 is considered optimal for written informational text (Clerehan & Buchbinder, 2006). To expedite our analysis, we used Linguistic Inquiry and Word Count software (Tausczik & Pennebaker, 2010), which can automatically count function words. We calculated the percentage of function words per 1,000-word section of each of the 14 documents. We took the inverse of this (i.e., subtracted from 100) to obtain the percentage of content words and divided this number by 10 to estimate lexical density (i.e., proportion of content words expressed on a scale from 0–10). For longer texts, we used the average of the 1,000-word sections.

### Factual Content

We inspected clinical guidelines for CKD lifestyle management (Kidney Disease: Improving Global Outcomes [KDIGO] CKD Work Group, 2013) to identify important information that should be included in the relevant materials. For self-management, clinical guidelines focus on exercise (30 minutes, 5 times per week), weight management (body mass index of 20–25 kg/m^2^), and smoking cessation. They recommend dietary advice be included in an education program tailored to CKD severity (i.e., stage), highlighting management of sodium/salt, phosphate, potassium, and protein intake. We considered whether this information was available in the materials. We also recorded the date the document was written, published, or reviewed, or the “review by” date if this was documented, to determine how current the information was likely to be.

### Format

Examination of format is included in the ELF for completeness, although it is not a linguistic consideration (Hirsh et al., 2009). We previously explored format and visual aspects using PEMAT-P (Shoemaker et al., 2013) and SAM (Doak et al., 1996) evaluation tools (Morony et al., 2017), and, therefore, did not repeat this using the ELF. For clarity, we present just a summary of those results as they apply to the 14 materials in this study.

## Results

**Table 2** summarizes the materials included in this analysis according to subject area (field), stated purpose (metadiscourse), the stated or implied readership (tenor), generic structure, the stage of CKD targeted, and mode of communication (brochure, magazine, poster, factsheet).

### Generic Structure

Across the 14 included materials, there appeared to be a potential (called a Generic Structure Potential) (Clerehan & Buchbinder, 2006) for 9 moves: (A) introducing the problem; (B) outlining tests and treatments; (C) instructing how to self-manage; (D) describing complications or related conditions; (E) working with health care professionals; (F) listing other information sources; (G) providing example documents; (H) considering psychosocial functioning and relationships; and (I) summarizing. One move (J) representing miscellaneous other moves (e.g. requesting donations) differed by field.

**Table 3** lists the moves that were present in the self-management and diet and nutrition materials at three levels: first, the moves (A, B, C); second, the rhetorical elements (1, 2, 3); and third, the topics related to the moves (i, ii, iii). Materials differed substantially on the inclusion of rhetorical elements and related topics within a move. Of the 9 identified moves only 2 moves, ([A] introducing the problem and [C] instructing how to self-treat or self-manage the condition), were common to all 14 materials; 1 move (F), referring to other information sources, was in 13 of the materials. The inclusion of moves is detailed in **Table A**. Introducing the problem was typically the first move, and giving referrals to other sources of information (F) was usually at the end of the document. We could not identify any other pattern to the sequence of moves. Many materials repeated moves, resulting in related information located in several different places. Under (E), materials typically referred patients to a dietitian for specific advice or encouraged them to speak to their doctor. Only 4 materials included a summary.

***Self-management.*** The 8 documents ranged in length from 1 to 30 pages. Two were part of a larger document and one was a poster. In addition to the two common moves (i.e., A and C), all materials included (D), which described complications or related conditions. Most (*n* = 7) documents also included (E) working with health care professionals, and (F) referring to other sources.

***Diet and nutrition.*** The 6 materials ranged from 2 to 32 pages, and all were stand-alone documents. Four moves were common to all diet materials: In addition to three common moves (A, C, F), all documents included (B), outlining medical tests and treatments.

### Rhetorical Elements

Typically, the materials served to define terms and inform the reader about their condition and/or strategies to self-manage. Instructions were frequently expressed using passive language (e.g., “Exercise regimes should be started gradually and professional advice is recommended especially if you have not exercised for a long time”) where the instructive purpose is not clear; or they simply suggested actions (e.g., “Most patients are advised to reduce their salt intake as it makes them thirsty”). This does not help patients to identify if they are in the category of “most” (and the statement could also be critiqued on a factual basis). Less frequently, materials instructed people explicitly (e.g., “Choose and prepare foods with less salt and sodium”). Many materials assumed that patients understood the meaning of what they needed to do (e.g., understanding what salt and sodium are and how to limit them, and what it means to “exercise regularly and avoid stress” and “maintain a healthy blood pressure”).

### Writer-Reader Relationship

**Table 2** shows that most materials were addressed to any patients with CKD. Some specified that they were focused on early CKD, whereas others discussed kidney failure and progression to dialysis or transplantation. All the diet and nutrition materials were addressed to people with CKD, whereas some of the self-management materials offered general advice that might be appropriate for anyone wanting to improve their (kidney) health, leaving it up to patients to guess if it applied to them. One self-management brochure identified the reader with an introductory table indicating all stages of CKD (defined by glomerular filtration rate), and the stages of CKD to which this particular material was relevant. This table was present in a suite of materials from the same organization, providing a quick reference for patients and clinicians as to the likely relevance for any given individual.

The writer was typically a medical CKD expert representing a doctor or organization. Six of the 14 documents clearly identified document authors/contributors by name, but did not always list their roles or qualifications. One listed qualifications of contributors but did not name them. In general, the tone of the documents tended to be authoritative, guarded, and distant, reflecting thinking that was formal and analytical rather than personal and specific.

### Metadiscourse

Only 7 of 14 materials provided a clear purpose for the text; 4 other materials implied it in the title or a subheading (**Table 2**). One text stated the purpose in small print on the rear cover. Disclaimers were present in 5 documents and tended to be written in small font and complex language.

### Signposting

All materials used headings. Examples of predictive headers included “What are the tests for CKD?” and “What can I do to treat my CKD?”, thus engaging the reader directly and identifying questions readers may want to ask. Less informative headers included “Introduction,” “General Lifestyle Tips,” and “Kidney Health Advice,” which gave little indication of the content to follow.

### Technicality of Vocabulary

As outlined previously (Morony et al., 2017), the language used in the CKD information materials was typically too discipline-related for the readership, which may include people other than the patient.

### Lexical Density

The lexical density across the 14 texts ranged from 5 to 6.8 (**Table A**). This is above the recommended range for written text (i.e., ≤5 [Clerehan et al., 2005]), suggesting that the information is dense and will require reader effort to “unpack.” For example, the sentence “*People* with a *long-term condition* such as *kidney disease* can *benefit* from being *involved* in and *taking responsibility* in their *own health* and *well-being*” has a lexical density of 5.4 (content words are in italics). By contrast, this sentence: “The *decisions* you *make* are not just to *please* your *doctor*, but for your *own benefit*” has a lexical density of 3.8. In a full text, higher-density sentences can make for weighty reading.

### Factual Content

Most materials (*n* = 11) gave a publication date. Most self-management materials identified the general need to exercise (*n* = 7), manage weight (*n* = 8), and quit smoking (*n* = 6), as required by the clinical guidelines KDIGO CKD Work Group, 2013); however, only one provided specific advice congruent with the guidelines (e.g., step-by-step advice for quitting smoking.) Furthermore, the rationale for adopting other lifestyle changes was not always clear.

All of the diet and nutrition materials covered the four diet elements, (sodium, potassium, phosphorous, protein), with most offering specific information. This is detailed in **Table A**. All mentioned the importance of “limiting” sodium intake, and getting the “right” amount of protein, but were vague about quantities; only one specified a maximum daily allowance of sodium. It was not always clear what this meant in terms of actions readers should take. Most recommended discussing with a health professional, which may not be feasible for many readers.

### Format

As outlined previously (Morony et al., 2017), we found that layout was acceptable overall. Materials typically followed recommended guidelines (Centers for Disease Control and Prevention, 2013; Shoemaker et al., 2013) for layout, meaning that most used headings, chunked text into sections, used bullet lists, and had plenty of white space. Pictures tended to be decorative rather than informative, however, and captions were frequently absent or unhelpful. Two materials included visual elements contradicting the text (i.e., photographs of foods to avoid that were not clearly marked as such [Morony et al., 2017]).

## Discussion

We explored key linguistic elements relevant to making meaning from patient education materials by applying the ELF to a set of CKD self-management and dietary information. We identified nine structural “moves” that were inconsistently used across the body of documents. This suggests a lack of awareness of the kind of structuring and sequencing that will be useful to users, making it clear what they want and need to know (see also Hirsh et al., 2009), and the (relative) importance of any actions they may need to take. Readers may include patients, family members, and caregivers, especially those responsible for meal preparation, who may be less familiar with CKD terminology. Trying to accommodate all people at once makes it difficult to deliver a clear and meaningful message (e.g., “Some people need to drink large amounts of fluids but others may need to limit their fluid intake”). This is consistent with prior analyses of “actionability” (Morony et al., 2017). It is not clear what advice such as “exercise regularly and avoid stress,” or “drink alcohol in moderation” means for a given reader, or how readers should quantify words such as “increase,” “limit,” “regularly,” and “moderation.” We suggest this advice needs to be more specifically worded, using appropriate rhetorical functions so the message is clear and unambiguous, such as “exercise for at least 30 minutes every day” (applicable to a particular stage), and include examples of suitable exercise.

The metadiscourse, which indicates the purpose of the text and how readers should approach it, was frequently inadequate, and legal disclaimers in the fine print advising the publisher could not guarantee adequacy of information have questionable usefulness for readers. The level of lexical density was generally high, suggesting that many readers would find the text too dense. Document developers can check lexical density by counting the number of content words on a given page and dividing it by the total number of words on the page.

Most leaflets on diet and nutrition make the assumption that all CKD patients have ready access to a dietitian, ignoring financial or logistical constraints. Given that the intended audience for these materials (i.e., people with CKD) may have low health literacy (Fraser et al., 2013; Lambert, Mullan, Mansfield, & Lonergan, 2015), education materials should make clear and consistent statements that are meaningful for them and easy to follow. Instead, we found the specific audience and their needs were often not clearly understood, and texts were usually not explicit regarding the stage of kidney disease or, in some cases, even the precise health condition for which the information was targeted.

In this study, we used ELF to analyze existing texts, but ELF can also guide development of texts through structured user-testing (Hirsh et al., 2009). The value of the ELF analysis is that specific strategies arise from the nine items and the assessment questions that enable document developers to create more reader-sensitive patient educational materials. Application of the ELF can help writers improve and diversify their texts and serve to inform clinicians' decision-making regarding suitable texts, thereby improving the matching of text to patient. For example, identifying structural elements and intended readership may help developers to focus on the most relevant content, as well as appropriate language and visual elements. CKD is a complex condition, and diet and self-management needs vary as it progresses. Some advice (e.g., “don't smoke”) will be relevant to all, whereas other advice (e.g., “limit fluids”) is dependent on disease stage. Prior work combining patient feedback with ELF analysis of medication information leaflets for rheumatoid arthritis (Hirsh et al., 2009), colorectal cancer screening kits (Fransen, Dekker, Timmermans, Uiters, & Essink-Bot, 2017), and decision aids (Smith, Trevena, Nutbeam, Barratt, & McCaffery, 2008) also suggested that information is not always aligned with what patients needed to know and do. This could be rectified by asking patients what they need to know, and referring to existing reports of user testing that identify patient priorities (KDIGO CKD Work Group, 2013).

### Limitations

Our analysis does not cover all materials within these topic areas because we specifically excluded online materials. Nevertheless, the substantial variation we identified across materials suggests that patient education providers may be assisted by a framework that can offer guidance as to how to best develop and present information to a diverse readership. A limitation of the study is that the sample of patient education materials in each category was small, and some of them appeared, without stating it, to be targeted more toward late-stage CKD (i.e., patients considering dialysis or transplantation). This reflected the current state of printed information materials on self-management for people with CKD. Comparing documents of different lengths and different modes or channels of communication (e.g., poster, brochure, section of a booklet) added to the variation in the structure of the texts themselves, making it difficult to identify the key elements that should be included for patient satisfaction.

## Conclusion

Patient information materials are an important adjunct to verbal interaction between health care practitioner and patient. Their value is dependent upon whether they contain useful information from the viewpoint of the patient and are trusted and understood. In this and previous studies (Morony et al., 2017), we found that CKD lifestyle advice is not always specific and actionable, so consumers may not know what to do with it. More importantly, it is often not clear at whom the material is targeted. The ELF has demonstrated value for analyzing and improving the quality of written patient information through both expert review (as in this study) and structured user-testing. As patient use of the internet and mobile phone apps advances, future research could investigate adapting the ELF's systemic functional approach for use on electronic materials.

## Figures and Tables

**Table 1:** The Evaluative Linguistic Framework for Health Care Text

**Item**	**Description**	**Assessment**
Overall organizational or generic structure	Series of “moves” in the text (e.g., background on disease or condition, elements of lifestyle to self-manage)	What identifiable moves are present? Are essential moves included? What is the sequence of moves and is this appropriate?
Rhetorical elements	The function of each move in relation to the reader (e.g., to define, to instruct, to inform)	What are the rhetorical functions of each move in relation to the reader? Are these clearly defined and appropriate? Is there clear guidance about what to do with the information? Are instructions clear about what action that needs to be taken?
Writer-reader relationship	Nature of the relationship of writer to reader (e.g., doctor to his/her patient; support organization to patient)	Is it clear who the writer is and who is the intended audience? Is the relationship between writer and reader clear and consistent? Is the person who is expected to take responsibility for actions clear? Is the importance and/or urgency of the action made clear? How positive (encouraging, reassuring) is the tone?
Metadiscourse	Description of the purpose/structure of the text Advertising, legal disclaimers, and other information about the text	Is there a clear description of the purpose of the text? Are disclaimers clear?
Signposts	Headings in printed text act as signposts for readers	Are headings present? If present, are they appropriate?
Technicality of vocabulary	Technicality/specialization of the medical terminology/other vocabulary	How technical is the vocabulary? Is it appropriate?
Lexical density	Density of the content words in the text	What is the average content density of the text (content-bearing words as a proportion of total words, expressed out of 10)? Is it appropriate (e.g., 3–4 or below if possible)?
Factual content of text	Facts included in the text	Is the factual information correct and up-to-date? Is the source of information provided? Is the quality and strength of the evidence discussed?
Format^a^	Visual aspects such as layout, font size, use of visual material	What is the length, layout, font size, and visual aspect of the document?

Note. Adapted from “A linguistic framework for assessing the quality of written patient information: Its use in assessing methotrexate information for rheumatoid arthritis” by R. Clerehan, R. Buchbinder, & J. Moodie, 2005, *Health Education Research*, 20(3), p. 334–344.

a Format included for completeness, although not a linguistic consideration.

**Table 2:** A Summary of Materials Included in This Analysis

							**Mode of Communication**		
**Field**	**Title (Year of Publication)**	**Organization**	**Author**	**Stated Purpose (Metadiscourse)**	**Intended Reader**	**CKD Stage**	**Number of Pages^a^**	**Type**	**Use of Graphics**
Self-management	*Living Well with Chronic Kidney Disease* (2010)	American Kidney Fund	Contributing doctors named in acknowledgment	To help you understand your CKD and what you can do to keep from getting worse	People whose doctor has told them “your kidneys are not working as well as they should” (i.e., who have CKD)	Early	19	Brochure	Photographs, diagrams, tables
	*How to Keep Your Kidneys Healthy* (2013)	British Kidney Patient Assocation	Named consultant (nephrologist)	In title	The general population	N/A	2	Factsheet	None
	*Looking After Yourself with Kidney Disease* (2013)	Kidney Health Australia	Not identified	In title	People who have been diagnosed with kidney disease	All	5	Factsheet	Photographs, proformas (e.g., table, graph)
	*Healthy Living for People with Kidney Disease* (2011)	Queen Elizabeth Hospital Birmingham (United Kingdom)	Named person (qualification not listed)	General and CKD-specific lifestyle advice for staying healthy and avoiding illness	People with kidney disease (all stages)	All	6	Brochure	None
	*You're in Charge* (unknown)	Kidney Health Australia	Named people representing organization (includes photos)	Help you to self-manage or avoid CKD	Someone with early or advanced kidney disease (or some other chronic illness) or their caregiver	All	9	Magazine	Photographs, health diary proforma
	*Living with Reduced Kidney Function* (2011)	Kidney Health Australia	Multiple contributors named in acknowledgements	In title	People (newly diagnosed) with early CKD	Early	26	Chapters	Cartoon illustrations
	*Following Your Treatment Plan* (2012)	Medical Education Institute (US)	Not identified	Outline key elements of treatment plan and provide tools to help you follow it	People with a CKD treatment plan	All	16	Learning module	Photographs
	*Chronic Kidney Disease: How to Look After Yourself* (unknown)	Kidney Health Australia	Not identified	In subheading	People with CKD	Not stated	1	Poster	Photographs, table, diagram
Diet and nutrition	*Nutrition and Kidney Disease* (2014)	Kidney Health Australia	Not identified	Not stated	People with kidney disease	Not stated	3	Factsheet	Photographs
	*Chronic Kidney Disease and Nutrition* (unknown)	Kidney Health New Zealand	Registered dietitians	Not stated	People with CKD	Not stated	10	Brochure	Photographs
	*Nutrition and Chronic Kidney Disease (Stages 1–4): Are You Getting What You Need* (2010)	National Kidney Foundation (US)	Not identified	Understanding nutrition for people with CKD	People with CKD	1–4	32	Brochure	Photographs
	*Kidney Failure and Healthy Eating* (2007)	Melbourne Health (Australia)	Authorized by named clinical director	Understanding healthy eating for those with kidney failure	People with kidney failure	5	2	Factsheet	Illustrations
	*Eating Right for Kidney Health: Tips for People with Chronic Kidney Disease* (2014)	National Kidney Disease Education Program (US)	Not identified	Tips to eat right for people with CKD	People with CKD	Early	4	Factsheet	Illustrations
	*Manage Your Chronic Kidney Disease (CKD Stages 3–4) with Diet* (2009)	Abbott Nutrition (US)	Not identified	In title	People with CKD	3–4	7	Brochure	Photographs

Note. CKD = chronic kidney disease; N/A = not applicable.

a Page size is A4/letter.

**Table 3:** Generic Structure: List of Moves, Number of Materials that Included the Moves, Rhetorical Functions, and Subtopics

	**Rhetorical Functions**			
**Moves**	**Self-Management**	***N***	**Diet and Nutrition**	***N***
A. Introducing the problem and why it is important	1. *Describing* function of kidneys 2. *Describing* CKD (including stages of CKD) 3. *Informing* why self-management is important 4. *Listing* risk factors for CKD	8	1. *Describing* function of kidneys 2. *Describing* CKD 3. *Informing* why diet/nutrition is important 4. *Explaining* that needs change as CKD progresses	6
B. Outlining (medical) tests and treatments	5. *Explaining* tests for CKD 6. *Describing* medications for CKD	6	5. *Describing* nutritional health checks 6. *Describing* medications for CKD	6
C. Instructing reader to self-manage	7. *Explaining* lifestyle elements reader needs to manage i. Diet – protein/potassium/sodium/phosphorous ii. Manage weight iii. Exercise iv. Don't smoke v. Diabetes/blood sugar vi. Blood pressure vii. Moderate alcohol viii. Manage fluids ix. Get immunized x. Manage medications xi. Sleep xii. Control cholesterol xiii. Protect from sun xiv. Things to avoid (e.g., NSAIDS, contrast dye) xv. Set goals xvi. Read food labels	8	7. *Explaining*elements of diet reader needs to manage i. Energy (calories) ii. Protein iii. Sodium iv. Phosphate v. Potassium vi. Fluids vii. Other nutrients 8. *Discussing* specific diets i. Diabetes ii. Vegetarian 9. *Discussing* weight management 10. *Reading* food labels	6
D. Describing complications or related conditions	8. *Describing* related conditions i. Diabetes ii. Blood pressure iii. Dialysis and ESRD	8	11. *Describing* related conditions i. Diabetes ii. Blood pressure iii. Dialysis and ESRD 12. *Explaining* that nutritional needs are individual and change over time	4
E. Working with health care professionals	9. *Advising* how to prepare or make the most of health care consultations 10. *Suggesting* questions to ask 11. *Defining* patient and health care team role/responsibilities 12. *Reminding* to inform health care professionals you have CKD	7	13. *Describing* how to work with a dietitian 14. *Suggesting* questions to ask	5
F. Listing other information sources	13. *Referring* to health care providers (not mentioned in E) 14. *Providing* glossary (instructing) 15. *Listing* source material/references 16. *Telling* personal stories/health care provider interviews 17. *Suggesting* additional resources 18. *Describing* how to evaluate websites	7	15. *Referring* to health care providers (not mentioned in E)	6
G. Providing example documents to complete	19. Health diaries 20. Medication checklists 21. Health care provider details	5	16. Food diaries 17. Notes 18. Question sheets to bring to doctor	2
H. Considering psychosocial functioning and relationships	22. *Informing* about caregiver rights and responsibilities 23. *Discussing* sexuality and intimacy 24. *Discussing* mental health 25. *Discussing* social support	3	19. *Encouraging* patient advocacy	1
I. Summary	26. *Summarizing* contents	3	20. *Summarizing* contents	1
J. Miscellaneous	27. *Requesting* donations 28. *Reassuring* that CKD is manageable	1	21. *Informing* about health care cover 22. *Discussing* dietary supplements and taking vitamins/minerals	3

Note. CKD = chronic kidney disease; ESRD = end-stage renal disease; NSAIDs = nonsteroidal anti-inflammatory drugs.

**Table A:** Summary of Materials and Scores Organized by Field

**Title**	**PEMAT**		**Language (Lexical Density)**	**Concordance with Clinical Guidelines**				**Inclusion of Moves (A–J) and Relationships to Rhetorical Elements**									
**Self-management**	**Undst.**	**Actnbl.**		**Exercise**	**Weight**	**Smoking**	**Diet Elements^a^**	**A**	**B**	**C**	**D**	**E**	**F**	**G**	**H**	**I**	**J**
*Living Well with Chronic Kidney Disease*	82	50	5.4	S	G	-	4	C	C	C	C	C	C	C	-	-	C
*Following Your Treatment Plan*	63	60	4.9	G	G	-	2	C	C	C	C	C	C	C	-	-	C
*Chronic Kidney Disease: How to Look After Yourself*	57	50	-	S^b^	G	S	0	C	-	C	C	C	C	-	-	-	-
*You're in Charge*	50	67	5.5	S^b^	G	S^b^	1	C	C	C	C	C	C	C	C	C	-
*Looking After Yourself with Kidney Disease*	44	50	5.3	S^b^	G	S^b^	3	C	C	C	C	C	C	C	C	-	-
*Healthy Living for People Living with Kidney Disease*	42	40	6.2	G	G	S^b^	1	C	-	C	C	I	C	-	-	-	-
*Living with Reduced Kidney Function*	35	57	5.2	S^b^	S^b^	S^b^	4	C	I	C	C	C	C	C	C	-	C
*How to Keep Your Kidneys Healthy*	15	40	5.2	-	G	S^b^	0	C	C	C	C	-	-	-	-	-	-
**Diet and nutrition**																	
*Eating Right for Kidney Health: Tips for People with Chronic Kidney Disease*	82	50	6.9	-	-	-	4^b^	C	I	C	-	I	C	I	-	-	-
*Chronic Kidney Disease and Nutrition*	71	50	5.8	-	-	-	4^b^	C	C	C	-	I	C	-	-	C	-
*Nutrition and Chronic Kidney Disease (Stages 1–4): Are You Getting What | You Need?*	65	33	5.7	-	G	-	4^b^	C	C	C	C	C	C	C	C	C	C
*Managing Chronic Kidney Disease (CKD Stages 3–4) with Diet*	53	33	5.6	-	-	G	4	C	C	C	C	C	C	-	-	C	-
*Kidney Failure and Healthy Eating*	47	20	5.6	-	-	-	4^b^	C	C	C	C	-	C	-	-	-	-
*Nutrition and Kidney Disease*	33	40	5.6	-	G	-	4^b^	C	C	C	C	C	C	-	-	-	-

Note. Actnbl. = actionability; C = consistent; G = generic advice; I = inconsistent; PEMAT = Patient Education Materials Assessment Tool; S = specific advice; Undst. = understandability.

a Number of key diet elements (sodium, potassium protein, phosphate).

b Specific advice consistent with guidelines.
